# Singular Configuration Analysis and Singularity Avoidance with Application in an Intelligent Robotic Manipulator

**DOI:** 10.3390/s22031239

**Published:** 2022-02-06

**Authors:** Helin Wang, Ziqiang Zhou, Xianyou Zhong, Qijun Chen

**Affiliations:** Department of Control Science and Engineering, Tongji University, Shanghai 201804, China; whl7228@tongji.edu.cn (H.W.); ziqiang.zhou@tongji.edu.cn (Z.Z.); zhongxianyou@tongji.edu.cn (X.Z.)

**Keywords:** co-bot, modeling robotic systems, kinetic inverse solution, singular configuration analysis, singularity avoidance, load and torque sensors

## Abstract

Recently, robotic sensor systems have gained more attention annually in complex system sense strategies. The robotic sensors sense the information from itself and the environment, and fuse information for the use of perception, decision, planning, and control. As an important supplement to traditional industrial robots, co-bots (short for co-working robots) play an increasingly vital role in helping small and medium-sized enterprises realize intelligent manufacturing. They have high flexibility and safety so that they can assist humans to complete highly repetitive and high-precision work. In order to maintain robot safe operation in the increasing complex working environment and human–computer intelligent interactive control, this paper is concerned with the problem of applicant accuracy analysis and singularity avoidance for co-bots. Based on the dynamic model with load and torque sensors, which is used to detect the external force at the end of the robot, this paper systematically analyzes the causes of singularity phenomenon in the robot motion control. The inverse solution is obtained by analytical method and numerical method, respectively. In order to ensure the smooth and safe operation in the whole workspace, it is necessary for a robot to avoid singularity. Singularity avoidance schemes are utilized for different control tasks, including point-to-point control and continuous path control. Corresponding simulation experiments are designed to verify the effectiveness of different evasion schemes, in which the advantages and disadvantages are compared and analyzed.

## 1. Introduction

Recently, appliance accuracy analysis and human–computer interaction have undergone rapid development in intelligent assembly machine factories, with typical examples including assembly, polishing, dual-arm coordination, or dexterous hand manipulations [1,2,3]. In order to adapt to small-batch, customized, and short-cycle production, it is necessary for co-bots, a new type of industrial robot that can work with human beings in the same working environment, to develop a hierarchical control algorithm that enables safe and stable cooperative locomotion. With dynamic model and torque sensors, the robot is achieving the drag control by compensations of gravity and friction, which counteract external disturbances. However, it will inevitably lead to the complexity and uncertainty of environment [4,5]. Reaching the singular region makes it easy for a robot to cause various problems, such as instability, poor performance, and so on. Emphasis is placed on the co-bot singularity. Hence, the singularity avoidance is the basis to ensure co-bot stable operation in the man–machine cooperation.

The singular configuration is a phenomenon which hampers the motion of the robot end effector [6,7]. The freedom of the robot end will decrease in a singular configuration, so it cannot be controlled to move in directions. While the robot approaches the singular adjacent region, some joints calculated by the inverse kinematic tend to infinity, which will cause the decline of tracking expected trajectory [8]. The singularity is the inherent characteristic of an articulated robot, which mainly appears in finding the inverse kinematics solution. The actual performance is that the calculated expected joint speed is much greater than the maximum joint speed which the actual motor can provide. Bohigas [9] gave the general algorithm of robot singular configuration with different mechanical structures. Müller [10] analyzed singular points by using Lie groups separated from the specific structure.

For more general singularity avoidance problems, Maciejewski [11] proposed a damped least square method with a damping term near singular region to obtain approximate solution of joint velocity. The essence lies in limiting the joint velocity near the singularity by adding a damping coefficient. Serial robots conduct seam welding, sealant application, spray painting, polishing, deburring, or other tasks requiring uninterrupted continuous motion. Chiaverini [12] introduced a continuous nonlinear function to characterize the damping factor, which is used to ensure the continuity of joint velocity near singular configuration. However, it will increase the tracking error of robot end in all directions. The damping factor is only added to the minimum singular value of a Jacobian matrix. In addition, Megalingam et al. [13] proposed singular separation to determine the singular direction of Kinova robots, and then avoid joint movement to pass through singular points. References [14,15] also proposed similar analysis methods to analyze singular directions, while these methods are not universal. A large number of SVD decomposition operations are required in the above singular point avoidance methods. In order to reduce amount of computation, Xu et al. [16] proposed the method of “singular separation + damping reciprocal” for PUMA robots. It was the first time to separate the singular problem into two parts of position and attitude. The redundant robots have infinite sets of inverse kinematics solutions, hence the optimal solution (i.e., the solution furthest away from the singular region) can be found by designing some optimization objective. Wang et al. [17] summarized some methods of using redundancy to avoid the singular region with series robot. The ultimate goal is to find the solution of the singular configuration of the robot principle in countless redundant solutions. The optimization directions include the determinant and condition number of Jacobian matrixes. However, the calculation using Jacobian matrix is too complex, and an optimization is proposed in reference [18,19].

All the singularity avoidance methods above are at the cost of tracking accuracy. The parameters of most methods are closely related to experience, especially the determination of damping factor and singular region. In addition to singularity avoidance method satisfying trajectory tracking, Taki et al. [20] proposed a method strictly tracking the desired path. Different from damping avoidance method, the avoidance algorithm proposed is to reduce running speed by reducing the expected speed when approaching the singular region, so as to avoid limiting the motion of the robot end effector caused by singular configuration. However, the strange avoidance method will only produce partial errors in tracking directions.

All these observations motivate the current study. In this paper, we concentrate on singular configuration analysis and singularity avoidance of co-bot, taking the JACO2 robot of Kinova Robotics company as an example [4,13]. It has seven DOF (degrees of freedom), with weight of 5.5 kg, a load of 1.5 kg, and a maximum arm span of 98.4 cm. Each joint motor is equipped with a torque sensor. The rotating potentiometer detects robot joint rotation, and the force sensor reflects ground reaction force information. The main contributions can be summarized as follows.

1. The D-H modeling method is utilized to build a joint coordinate system of the JACO2 robot. The inverse kinematics is solved by analytical method and numerical method, and the operation speed and accuracy of two schemes are compared.
2. The singularity caused by inverse kinematics is analyzed, and the robot singular configuration conditions are based on the block analysis of wrist Jacobian matrix, which are divided into three singular types: internal singularity, external singularity, and wrist singularity.
3. According to different task requirements, singularity avoidance schemes are utilized in robot redundancy, damped least squares, and singularity consistency. It is noted that these works focus on the working principle and control methods of robotic sensor system.

## 2. Materials and Methods

### 2.1. D-H Model

**Figure 1:**
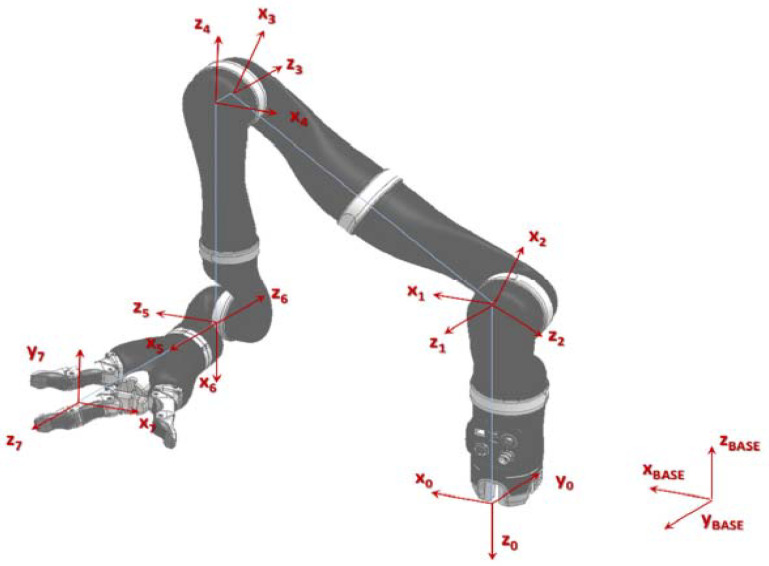
JACO2 robot coordinate system.

The kinematics model of the JACO2 robot with D-H model is illustrated in Figure 1, and its parameters are shown in Table 1.

According to D-H parameters, the transformation matrix between each links is

$${}^{\mathrm{base}}T{}_{0}=\left[\begin{array}{cccc} 1 & 0 & 0 & 0\\ 0 & -1 & 0 & 1\\ 0 & 0 & -1 & 0\\ 0 & 0 & 0 & 1 \end{array}\right],{}^{i}T{}_{i+1}=\left[\begin{array}{cccc} cos\theta_{i+1} & 0 & sin\theta_{i+1} & 0\\ sin\theta_{i+1} & 0 & -cos\theta_{i+1} & 0\\ 0 & 1 & 0 & d_{i+1}\\ 0 & 0 & 0 & 1 \end{array}\right],\;{}^{6}T{}_{7}=\left[\begin{array}{cccc} cos\theta_{7} & sin\theta_{7} & 0 & 0\\ sin\theta_{7} & -cos\theta_{1} & 0 & 0\\ 0 & 1 & -1 & d_{7}\\ 0 & 0 & 0 & 1 \end{array}\right],$$

where i=0,1,2…5. Thus, the forward kinematics equation of the JACO2 robot can be expressed as

(1) Tbasen=Tbase0T01T12T23T34T45T56T67=f(Θ)

where Tij denotes the homogeneous transformation matrix from {i} to {j} coordinate system, and $\Theta =\left\{\theta_{1},\theta_{2}\cdots \theta_{n}\right\}$ denotes angle information of each joint. The Euler angle (x-y-z)[α,β,γ] and $p={\left[p_{x},p_{v},p_{z},\alpha ,\beta ,\gamma \right]}^{T}$ denote attitude of robot end and end pose, respectively, where $\left[p_{x},p_{y},p_{z}\right]\;$ denote robot end position.

### 2.2. Inverse Kinematics

The analytical solution and numerical solution are generally adopted to resolve robot inverse kinematics. The former uses a robot’s geometric configuration by separating the forward kinematics parameters. The latter is based on numerical iteration, which obtains the optimal solution by setting objective function and controlling joint angle to move to the opposite gradient direction.

The JACO2 robot is a seven-DOF spherical revolution spherical configuration series robot. For a certain end pose, the robot has countless sets of inverse kinematics solutions. The redundancy makes the JACO2 robot operate more flexible than a traditional six-DOF series robot but increases the computational complexity. Two methods are used to solve inverse kinematics, respectively.

1. Analytical solution

The JACO2 robot has one more rotating joint at third joint and one more offset at fourth joint. Taking the third joint angle $\theta_{3}$ as the redundant parameter, other joint angles are

(2) $$\theta_{i}=f\left(\theta_{3}\right)\;\;\;\;\;\;\;i=1,2,I,7$$

All inverse kinematics solutions can be obtained by traversing all effective value, and only one set of optimal solution can be selected according to cost function. Therefore, the inverse kinematics optimization problem is simplified as

(3) $$\begin{array}{l} \min f\left(\Theta \right)\\ s.t.\;\;{}^{base}T\left(\Theta \right)={}^{base}T{}^{d}\;,\;\;\Theta \in \left[\Theta^{\min},\Theta^{\max}\right], \end{array}$$

where $\;\Theta ={\left[\theta_{1}\;\;\theta_{2}\;\;\theta_{3}\;\;\theta_{4}\;\;\theta_{5}\;\;\theta_{6}\;\;\theta_{7}\right]}^{T}$ is angle vectors of each joint, Tbase(Θ) is the forward kinematics equation, Tbased is the given end pose, and $\left[\Theta^{\min},\Theta^{\max}\right]$ represents the constraint of each joint.

Substituting (2) into (3), the inverse kinematics optimization problem is turned into a one-dimensional optimization of $\theta_{3}$:

(4) $$\min\;f\left(\theta_{3}\right)\;\;s.t.\;\Theta \in \left[\Theta^{\min},\Theta^{\max}\right].$$

Selecting the motion amplitude of each joint as the cost function,

(5) $$f\left(\Theta \right)=\sum_{i=1}^{7}\lambda_{i}{\left(\theta_{i}^{t+1}-\theta_{i}^{t}\right)}^{2},$$

where $\lambda_{i}$ is the weight coefficient, $\theta_{i}^{t+1}$ is the solution of the *i*-th joint in the current period, and $\theta_{i}^{t}$ is the solution of the *i*-th joint in the previous cycle. A greedy search is used to find the optimal solution.

2. Numerical solution

The numerical method focuses on the equation relationship instead of robot geometric configuration. Given this, f(Θ)=Tbasen, expected position Tbased, which are to be solved with constraints $\Theta \in \left[\Theta^{\min},\Theta^{\max}\right]$. The Jacobian matrix describes the relationship between robot end velocity and joint velocity:

(6) $$\dot{x}=J\dot{q}$$

where $\dot{x}={\left[v_{x}\;v_{y}\;v_{z}\;w_{x}\;w_{y}\;w_{z}\right]}^{T}$ represents terminal velocity. $R_{3\times 3}$ describes the relationship between Euler angle and terminal angular velocity.

(7) $$\dot{p}=\left[\begin{array}{c} I_{3\times 3}\\ R_{3\times 3}{}^{-1} \end{array}\right]\;\dot{x}=\left[\begin{array}{c} I_{3\times 3}\\ R_{3\times 3}{}^{-1} \end{array}\right]\;J\dot{q}=J’\dot{q},\;R_{3\times 3}=\left[\begin{array}{c} 1\;\;\;\;\;0\;\;\;\;\;\;\;\;\;\;\;sin\beta \\ 0\;\;cos\alpha \;\;-sin\alpha cos\beta \\ 0\;\;sin\alpha \;\;\;\;\;cos\alpha cos\beta \end{array}\right]$$

The pseudocodes of analytical and numerical inverse kinematics solutions are shown in Algorithms 1 and 2.
**Algorithm 1: According to the Cost Function (7), the Obtained Optimal Solution** 
$\theta_{3}$
**Input:**$T^{d}$ (End pose to be solved)$\Theta^{0}$ (Current joint angle vector)n (Iterative numbers per layer loop)ε (Minimum allowable error)$\left[\theta_{3}^{\min},\theta_{3}^{\max}\right]$ (Possible value range)**Output:**$\Theta^{opt}$ (Optimal joint angle vector)1:**do**2:  $r=\left(\theta_{3}^{\max}-\theta_{3}^{\min}\right)/\left(n-1\right)$ (Search step of $\theta_{3}$)3: 
$S=\left\{\theta_{3}^{\min}+\left(m-1\right)r|m=I\ldots n\right\}$
4:  $C_{\max}=inf$ (Cost)5:  for r in S do6:   
$\theta_{3}=r$
7:   $\Theta =InvKin\;\left(T^{d},\theta_{3}\right)$ (Given $\theta_{3}$ to find inverse kinematics solutions. If the    solution is within the joint limit, Θ  is returned; otherwise, Φ is returned.)8:   if Θ≠Φ and $f\left(\Theta \right)<C_{\max}$ then9:     
$C_{\max}=f\left(\Theta \right)$
10:     
$\Theta^{opt}=\Theta$
11:   else12:    continue13: 
**end**
14: 
$\theta_{3}^{\min}=\theta_{3}^{opt}-s$
15: 
$\theta_{3}^{\max}=\theta_{3}^{opt}+s$
16:**while** γ>ε


**Algorithm 2**
**:**

φ

**is the Partial Derivative of the Joint Angle**
**Input:**$P^{d}$ (Terminal position vector needed to be solved)$\Theta^{0}$ (Current joint angle vector)λ (Step size per iteration)$n_{\max}$ (Maximum number of iterations)ε (Maximum allowable error)**Output:**$\Theta^{opt}$ (Optimal joint angle vector)1:$\Theta =\Theta^{0}$,n=12:

$err=P^{d}-f\left(\Theta \right)$

3:
***do***
4:  $\Delta \Theta =J^{+}\cdot err+\left(I_{7\times 7}-J^{+}J\right)\;\varphi$
**(**φ is used to control the iteration direction)5:  ΔΘ=normalize(ΔΘ)
**(**Normalized to [-pi,pi])6:  
Θ=Θ+λ⋅ΔΘ
7:  
$err=P^{d}-f\left(\Theta \right)$
8: 
n=n+1
9:***while*** ‖err‖<ε or $n>n_{\max}$10:

$\Theta^{opt}=\Theta$



### 2.3. Singular Configuration Analysis

Both of the two methods illustrated in Section 2.2 will introduce singularity. Whether the robot is in a singular configuration depends on the full rank of the Jacobian matrix,

(8) $$J=\left[\begin{array}{c} J_{p}\\ J_{\omega } \end{array}\right]=\left[\begin{array}{c} v\\ \omega \end{array}\right]=\left[\begin{array}{c} e_{1}\times r_{1}\;\;e_{2}\times I\;\ldots \;e_{7}\times r_{7}\\ \;\;\;e_{1}\;\;\;\;\;\;\;e_{2}\;\;\;\;\;\;\;\;\;e_{7}\; \end{array}\right]$$

where $e_{i}$ is the unit vector of the i-th joint, and $r_{i}$ is the vector between the origin and end position. The Jacobian matrix at the end and wrist satisfies

(9) $$J_{e}=\left[\begin{array}{c} I_{3\times 3}\;\;U_{3\times 3}\\ 0_{3\times 3}\;\;I_{3\times 3}\; \end{array}\right]\;J_{w},\;U_{3\times 3}=\left[\begin{array}{c} \;\;0\;\;\;\;\;\;\;-d_{7}c_{6}\;\;\;\;\;-d_{7}s_{5}s_{6}\\ \;d_{7}c_{6}\;\;\;\;\;\;\;\;\;0\;\;\;\;\;\;\;\;\;\;d_{7}c_{5}s_{6}\\ d_{7}s_{5}s_{6}\;\;-d_{7}c_{5}s_{6}\;\;\;\;\;\;\;\;0\;\;\; \end{array}\right]\;.$$

Let $c_{i}$ and $s_{i}$ be abbreviations of $cos\theta_{i}$ and $sin\theta_{i}$, respectively, and that $det(J_{e}J_{e}^{T})=det(J_{w}J_{w}^{T})$, $Rank\left(J_{e}\right)=Rank\left(J_{w}\right)$. According to (8), the Jacobian matrix of wrist is

(10) $$J_{w}=\left[\begin{array}{c} J_{11\;3\times 4}\;\;\;\;\;0_{3\times 3}\\ J_{21\;3\times 4}\;\;\;J_{22\;3\times 3} \end{array}\right].\;$$

It is expressed as (11) in the wrist coordinate system:

(11) Jww=[Rbasew−1     03×3    03×3   Rbasew−1]baseJw

where Rbasew represents wrist Jacobian matrix.

1. Positional singularity

The unfilled rank of the first three rows of the Jacobian matrix will result in position singularity, which can be described as det(Jw11Jw11T)=0,

(12) det(Jw11Jw11T)=∑i=14Mi2

where $M_{i\;}$ represents the determinant of cofactor of matrix Jw11.

$$\begin{array}{l} M_{1}=det\left(\left[j_{1}\;j_{2}\;j_{3}\right]\right)=0,\\ M_{2}=det\left(\left[j_{1}\;j_{2}\;j_{4}\right]\right)=d_{3}d_{5}s_{4}\left(d_{3}s_{2}-d_{5}s_{2}c_{4}+c_{2}d_{5}c_{3}s_{4}+c_{2}d_{4}s_{3}\right),\\ M_{3}=det\left(\left[j_{1}\;j_{3}\;j_{4}\right]\right)=d_{3}d_{5}s_{2}s_{4}\left(c_{3}d_{4}-d_{5}s_{3}s_{4}\right),\\ M_{4}=det\left(\left[j_{2}\;j_{3}\;j_{4}\right]\right)=d_{3}d_{5}s_{4}\left(d_{4}s_{3}+c_{3}d_{5}s_{4}\right). \end{array}$$

The conditions in whether each co-factor equals zero are expressed as Table 2.

**Figure 2:**
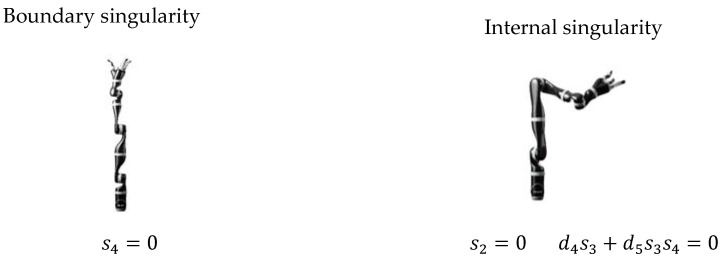
Position singularity of the JACO2 robot.

When $s_{4}=0$, the $\theta_{4}$ joint limitation is 32°~328°, the robot arms stretch out, and the end is at the boundary point, which is called boundary singularity. The $\theta_{2}$ joint limitation is 49°~311°, when $d_{4}s_{3}+d_{5}s_{3}s_{4}=0$, the JACO2 robot is in a singular state, which is called internal singularity, which is shown in Figure 2. The singularity condition is illustrated in Table 3.

2. Attitude singularity

**Figure 3:**
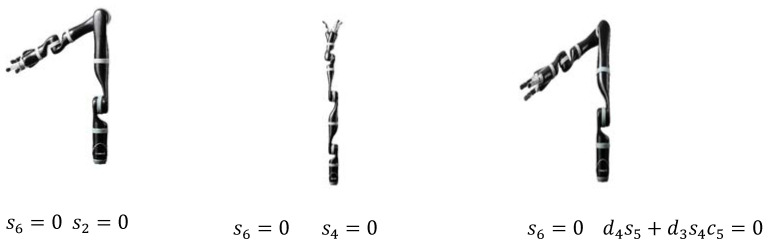
JACO2 wrist singularity.

Similarly, the attitude singularity can be judged by analyzing whether the last three rows of the wrist Jacobian matrix are less than the rank. Substituting each item of Jacobian matrix, the robot attitude singularity condition is shown in Table 4, and its wrist singularity condition is shown in Figure 3.

det(Jw22Jw22T)=s62=0,  det(JwwJwwT)=2d32d52s22s42(d4s5+d3s4c5)2=0.

## 3. Theoretical Analysis

The main purpose of singularity avoidance is to keep the stable, continuous, and bounded running speed of each joint when the robot is in the singular region. The relationship between joint velocity and end velocity is described as

(13) $$\dot{q}=J^{-1}\dot{x}$$

Since the Jacobian matrix of the JACO2 robot is a nonsquare matrix, (13) is turned into

(14) $$\dot{q}=J^{+}\dot{x}+\alpha \left(I-J^{T}J^{+}\right)\;\nabla \varphi$$

where $J^{+}=J^{T}{\left(JJ^{T}\right)}^{-1}$ represents the pseudo-inverse of the Jacobian matrix J, α is coefficient, and ∇φ is an arbitrary vector. In order to ensure that the joint velocity is bounded, it is necessary to change Jacobian matrix J or end running velocity $\dot{x}$.

### 3.1. Avoidance Strategy with End Velocity Constant

1. Internal singularity

To keep the end velocity constant, according to (15), only the Jacobian matrix *J* needs to be changed. The cost function (10) is introduced to obtain the optimal solution. The function is designed:

(15) $$f\left(\Theta \right)=\frac{1}{\sqrt{det\left(JJ^{T}\right)}}$$

where J  respresents Jacobian matrix corresponding to each group of possible inverse kinematics solutions. Then, $det\left(JJ^{T}\right)$ will be maximized to avoid singularity. The cost function can be designed as

(16) $$f\left(\Theta \right)=\frac{1}{\sigma_{r}^{2}}$$

(17) $$f\left(\Theta \right)=\frac{\sigma_{r}^{2}}{\sigma_{1}^{2}}$$

where $\sigma_{r}$ and $\sigma_{1}$ represent minimum and maximum singular value, respectively. The convergence direction can be controlled by selecting an appropriate vector ∇φ, which controls the gradient of cost function with respect to joint angle Θ. Hence, the equivalent effect of avoiding singularity with the analytical rule is

(18) $$\nabla \varphi =\frac{\partial f\left(\Theta \right)}{\partial \Theta }$$

However, considering that all redundant solutions of the JACO2 robot make the manipulator elbow operate on a spatial circle, the above avoidance method can only have a good avoidance effect on the internal singularity and yet have nothing to do with external singularity and pose singularity.

2. Other singular configurations

Other singular configurations cannot be simply avoided by redundant solutions. When the robot elbow is extended, which belongs to external singularity, the robot end is close to the workspace boundary. The robot cannot find the solution far away from the singularity (the fourth joint angle of all solutions approaches π). For six-DOF series industrial robots (most are PUMA type), the method of “singular separation + damping coefficient” adopted in literature [16,21] can obtain good results and analyze the corresponding errors, while it is not suitable for the JACO2 redundant robot, since singular separation effect cannot be realized directly. A damped least squares method is adopted for singular avoidance in this section, which combines the merit of Newton and steepest descent method. It not only ensures the convergence of iterative calculation, but also speeds up the convergence speed.

(19) $$\dot{q}=J^{\#}\dot{x}$$

where $J^{\#}$ is the pseudo-inverse of damped Jacobian matrix,

(20) $$J^{+}=J^{T}{\left(JJ^{T}+\lambda^{2}I\right)}^{-1}$$

Then, minimizing the cost function, and regularizing velocity term by coefficient,

(21) $$f\left(\Theta \right)=\sum_{i=1}^{7}{\left(\theta_{i}^{t+1}-\theta_{i}^{t}\right)}^{2}+\sum_{i=1}^{7}\lambda^{2}{\left({\dot{\theta }}_{i}^{t+1}\right)}^{2}$$

Then, we regularize of velocity term by coefficient. The minimum singular value of the Jacobian matrix is used as the criterion to judge whether the robot enters the singular region. Equation (20) can be transformed into

(22) $$J=\sum_{i=1}^{6}\sigma_{i}u_{i}v_{i}$$

(23) $$J^{\#}=\overset{6}{\underset{i=1}{\sum }}\frac{\sigma_{i}}{\sigma_{i}^{2}+\lambda^{2}}v_{i}u_{i}^{T}$$

(24) $$\dot{\Theta }=J^{\#}\dot{x}=\overset{6}{\underset{i=1}{\sum }}\frac{\sigma_{i}}{\sigma_{i}^{2}+\lambda^{2}}v_{i}u_{i}^{T}$$

$$\mathrm{where}\;\lambda^{2}=\{\begin{array}{c} \;\;\;\;\;\;\;\;\;0\;\;\;\;\;\;\;\;\;\;\;\;\;\;\;\;\;\;\sigma_{6}>\varepsilon \\ \left(1-{\left(\frac{\sigma_{6}}{\varepsilon }\right)}^{2}\right)\lambda_{m}^{2}\;\;\;\;\;\;\;\;\;\sigma_{6}\leq \varepsilon \end{array}$$

### 3.2. Avoidance Strategy with End Velocity Changed

The external singularity and wrist singularity are mainly discussed here. The avoidance strategy of end speed change is to change the expected end speed in (15). A path parameter *p* is introduced to describe the trajectory, and end operation trajectory can be expressed as

(25) x=x(p)

The tracking of desired trajectory is expressed by mathematical expression,

(26) $$h\left(\tilde{q}\right)=k\left(p\right)-x\left(p\right)=0$$

where k(p) represents the forward kinematics solution process, $\tilde{q}$ is the set of path parameters p and joint angles, i.e., $\tilde{q}=\left(q,p\right)$. The tracking target will satisfy

(27) $$dh\left(\tilde{q}\right)=H\left(\tilde{q}\right)d\left(\tilde{q}\right)=0$$

(28) $$H\left(\tilde{q}\right)=\frac{\partial h\left(\tilde{q}\right)}{\partial \tilde{q}}=\left[J\left(q\right)\;\;\;-S\right]$$

where S is a unit vector, which satisfies dx(p)=dpS, and ‖dv‖=‖dv(x)‖ direction, *S* is the vector of the end running speed, and parameter *P* is the end running speed. The general solution of (27) can be expressed as

(29) $$d\tilde{q}=bf\left(\tilde{q}\right)$$

where *b* is constant, $f\left(\tilde{q}\right)\in \ker\;H\left(\tilde{q}\right)$,

(30) $$f\left(\tilde{q}\right)={\left[f^{T}\left(q\right)\;\;\;detJ\left(q\right)\right]}^{T}$$

where $f\left(\tilde{q}\right)=\left(adjJ\left(q\right)\right)S\left(p\right)$. Equation (29) can be expanded to

(31) $$dq=bf\left(\tilde{q}\right)$$

(32) dp=bdetJ(q)

As long as the trajectory parameters are designed to satisfy (32) and give the joint speed of formula (31), the robot end trajectory can be always followed. It can be found that when the robot approaches the singular configuration, the determinant of the Jacobian matrix approaches 0, and the given constant and joint velocity both tend to infinity. Therefore, the singular phenomenon can be avoided. The constant *b* is determined by

(33) $$b^{*}=\frac{dq_{\max}}{f\left(\tilde{q}\right)}$$

Reprogramming end velocity based on (32),

(34) $$dp^{*}=b^{*}det\;J\left(q\right)$$

In that the JACO2 robot has redundant joints, (31) is deformed as

(35) $$dq=b_{ep}n_{ep}+b_{sf}n_{sf}$$

(36) $$dp=b_{ep}det\;J_{3}$$

where $b_{ep}$ and $b_{sf}$ are all constants, $J_{3}$ is the Jacobian matrix removing cofactor of column 3, $n_{ep}\in rowJ$, $n_{sf}\in \ker J$. However, when internal singularity occurs, the symbol of $det\;J_{3}$ and dp will change, making the expected trajectory reverse, which is equivalent to an obstacle.

(37) $$n_{ep}=\sum_{i=1}^{7}b_{i}n_{i}$$

(38) $$dp=b_{ep}\sum_{i=1}^{7}b_{i}det\;J_{i}$$

where $J_{i}$ is the Jacobian matrix removing the cofactor of column $i_{}$, the ith item of $n_{i}$ is always 0, then remove $n{’}_{i}$ item, there is $Jn{’}_{i}=C_{i}det\;J_{i}$. The ith item of $C_{i}$ is 1, and other items are 0.

Let $b_{i}=det\;J_{i}$, then $dp=b_{ep}\overset{7}{\underset{i=1}{\sum }}b_{i}det\;J_{i}=b_{ep}det\left(JJ^{T}\right)$, then the symbol dp will not change.

## 4. Experimental Results and Comparisons

To demonstrate the performance of the proposed algorithm, a series of simulations and comparisons are conducted. The simulations include a variety of avoidance strategies which basically cover most of the actual situations. Four experimental studies including inverse kinematics solutions and singularity analysis are carried out to test the feasibility of practical application. The symbolic operation toolbox in MATLAB is used for dynamic modeling.

The robot home position is selected as the starting point (joint angle [283.24; 162.71; 0.; 43.58; 265.23; 257.52; 288.14], pose position [0.2113; −0.2656; 0.5065; 1.6477; 1.1081; 0.1282]).

### 4.1. Two Inverse Kinematics Solutions

**Figure 4:**
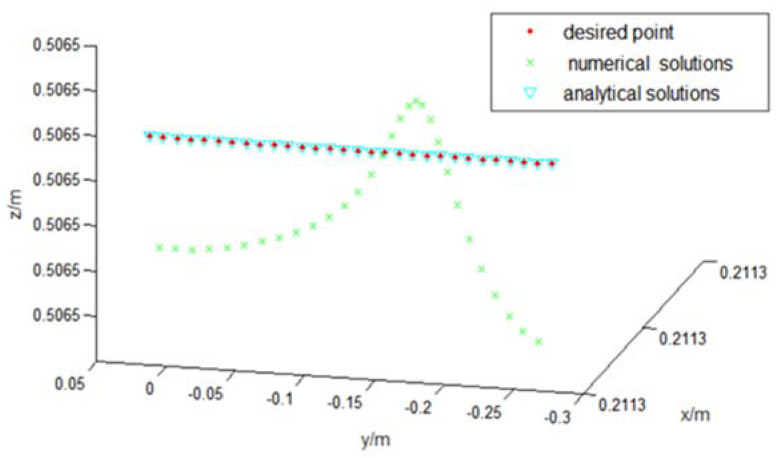
Comparisons of two inverse kinematics solutions.

**Figure 5:**
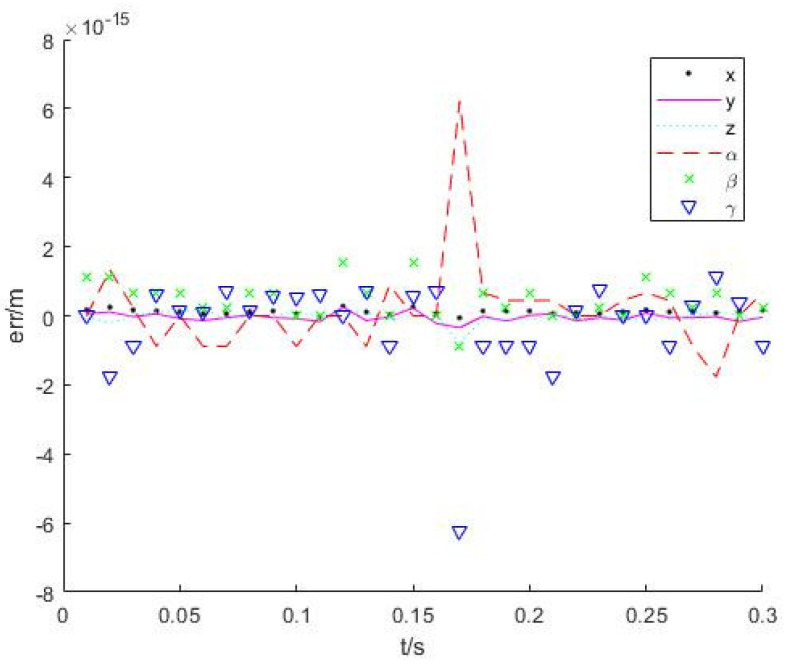
Analytical inverse solution error.

**Figure 6:**
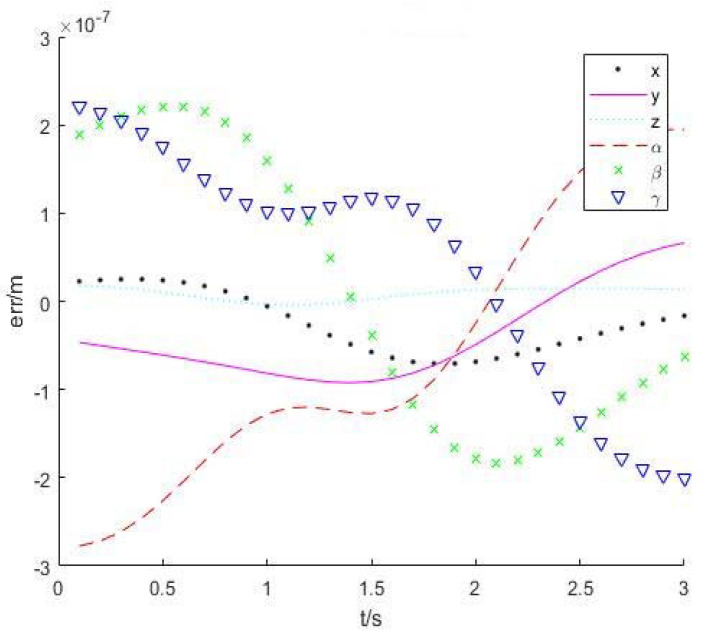
Numerical inverse solution error.

Let the robot end run at a speed of 0.1 m/s along the *y*-axis for 3 s, and control period be 0.1 s. A total of 200 trajectory middle points are inserted. The angles of each joint are calculated by two inverse kinematics solution algorithms. Figure 4 shows the trajectories of two kinematics algorithms. The red points represent the desired points, and blue and green describe trajectories with numerical and analytical solutions, respectively. Their solution errors are shown in Figure 5 and Figure 6.

### 4.2. Singularity Avoidance

To verify the effectiveness proposed in Section 3, singularity avoidance strategies based on robot redundancy, damped least squares, and singularity consistency are realized, respectively. Let the robot end run at the speed of 0.1 m/s along the positive direction along the *z*-axis for 7.5 s and then turn back, and continue to run along the negative direction along the *z*-axis for 7.5 s. Set a control cycle as 0.01 s. The joint state and tracking error are plotted with a sampling period of 0.1 s. The compared simulations are as follows.

1. Taking no avoiding measures

**Figure 7:**
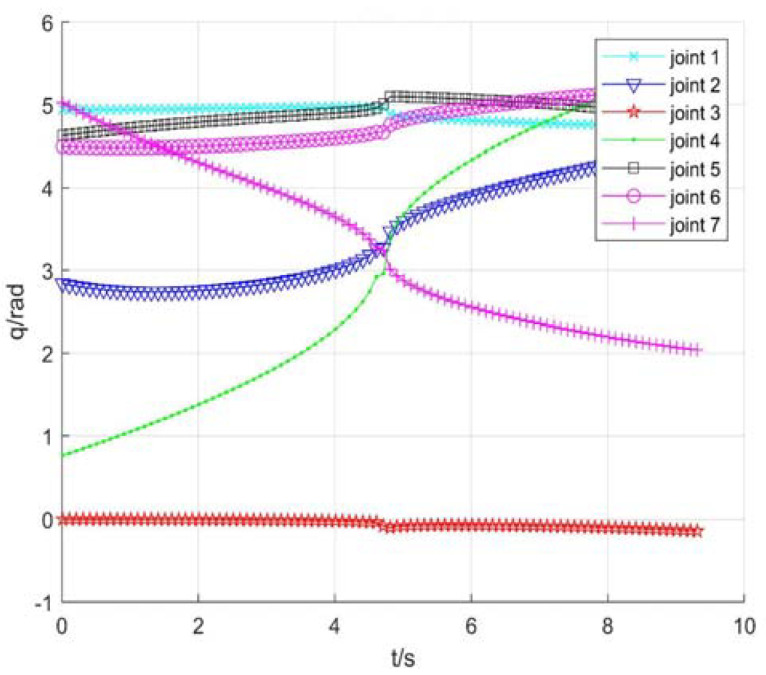
Position of each joint.

**Figure 8:**
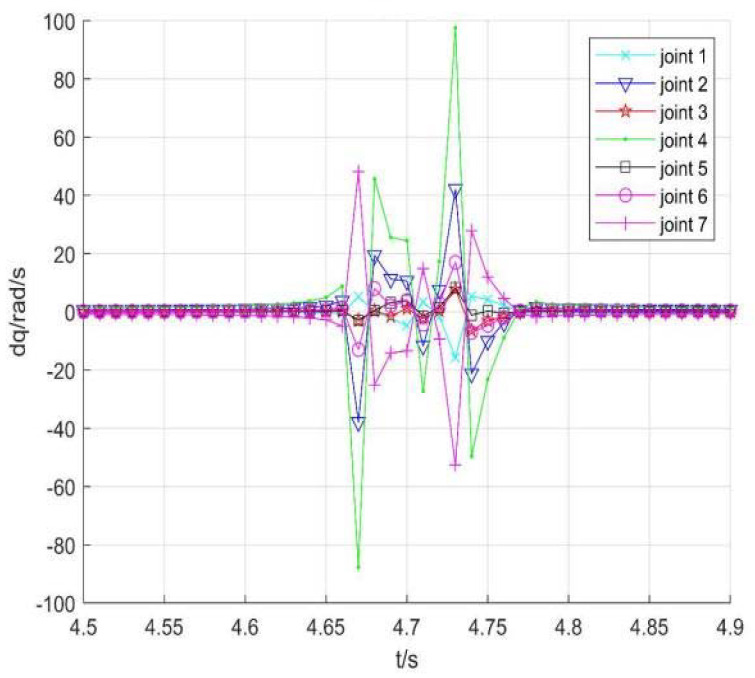
Velocity of each joint.

**Figure 9:**
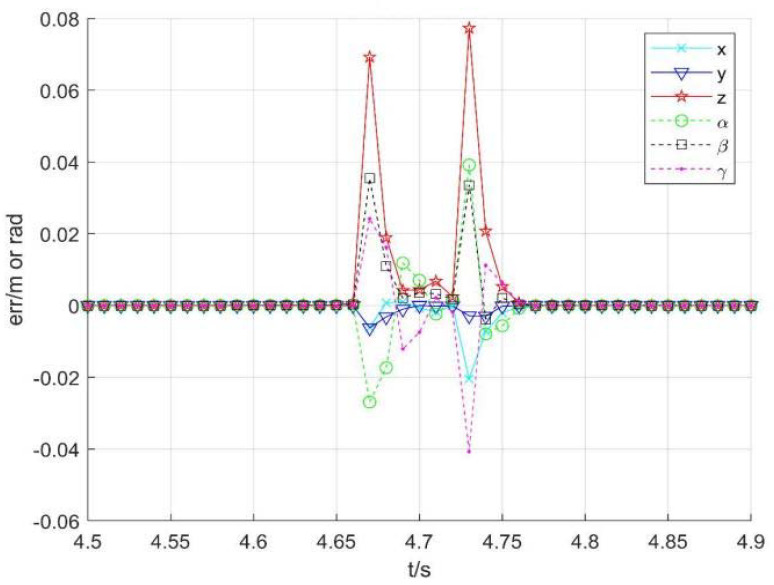
The tracking errors of position and orientation.

Figure 7 shows the position change curve of each joint, Figure 8 shows the velocity variation curve (4.5 s~4.9 s), and Figure 9 shows the tracking errors of position and orientation (4.5 s~4.9 s).

2. Using damping Jacobian matrix to avoid singularity

**Figure 10:**
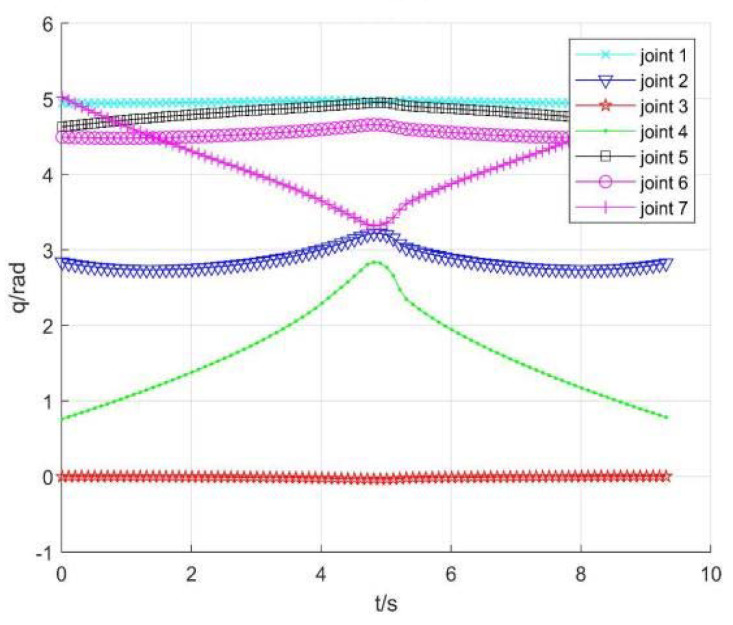
Position of each joint.

**Figure 11:**
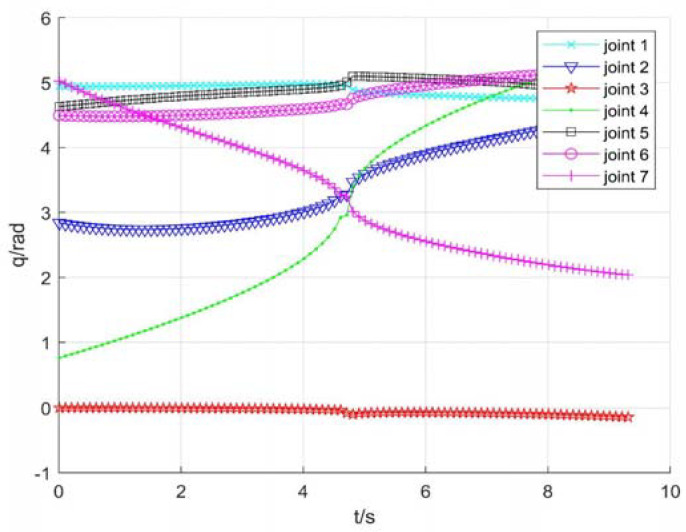
Velocity of each joint.

**Figure 12:**
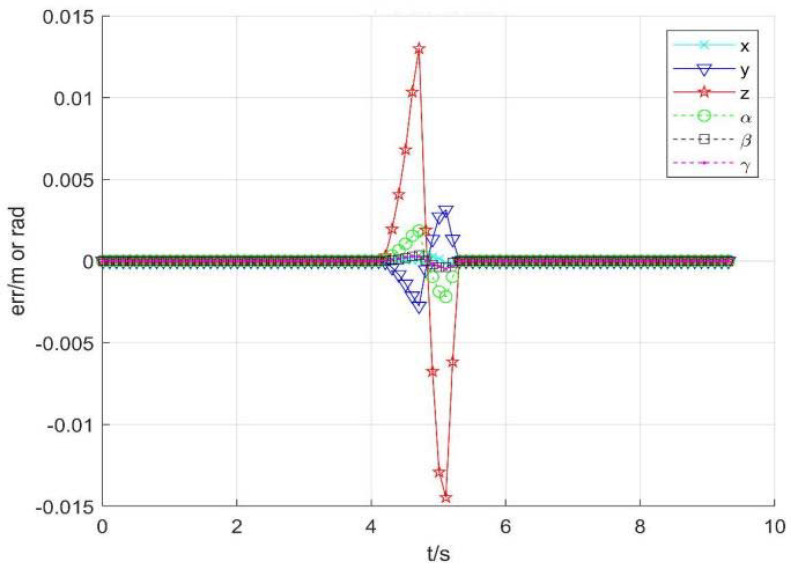
The tracking errors of position and orientation.

Assume that ε=0.1 is the boundary of the singular region, and $\lambda_{m}^{2}=0.1$ is the damping coefficient. Figure 10 shows position change curve of each joint, Figure 11 shows the speed change, and Figure 12 shows tracking errors of position and orientation, respectively.

3. Using singular consistency to avoid singularity

**Figure 13:**
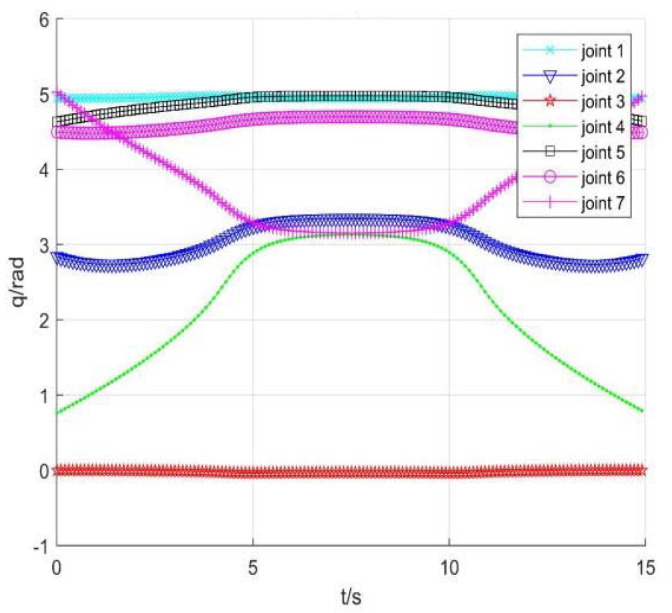
Position change of robot joints.

**Figure 14:**
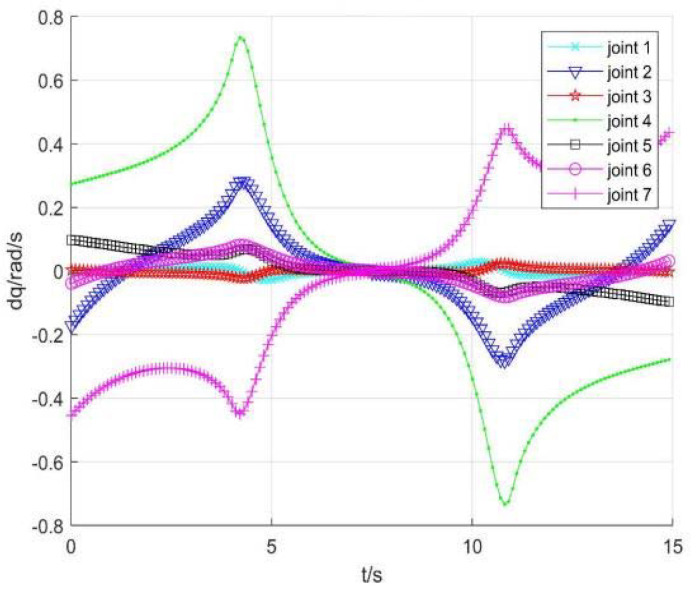
Velocity variation of robot joints.

**Figure 15:**
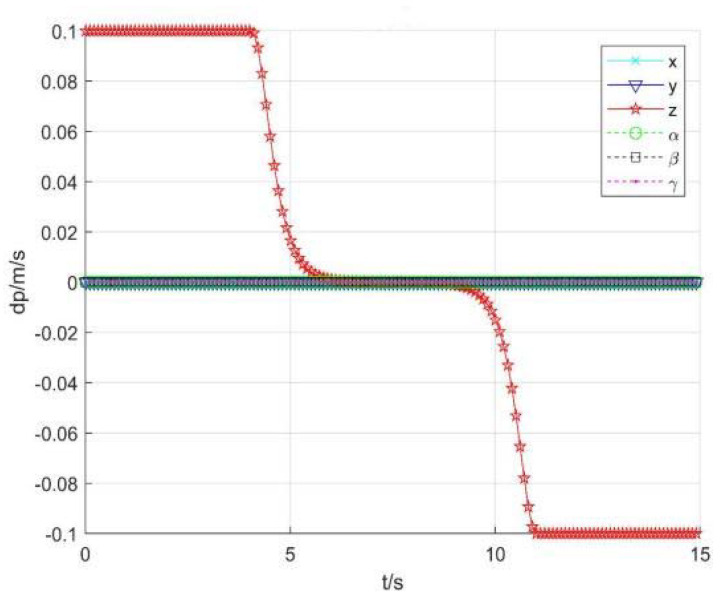
Robot end speed change.

**Figure 16:**
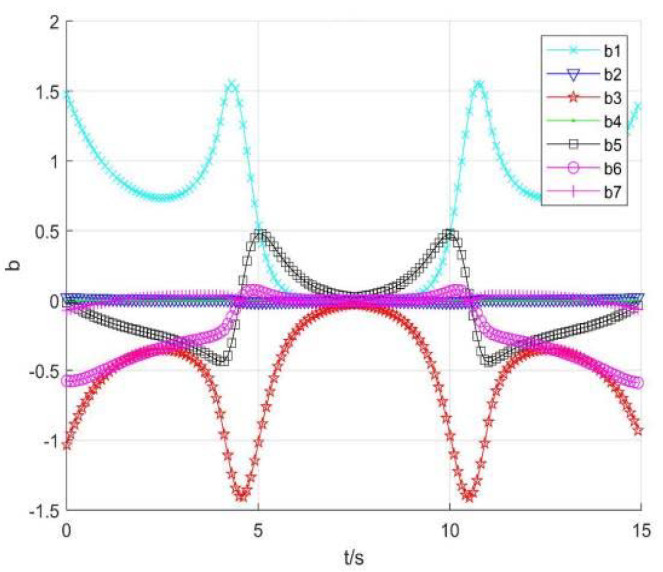
The constant *b.*

**Figure 17:**
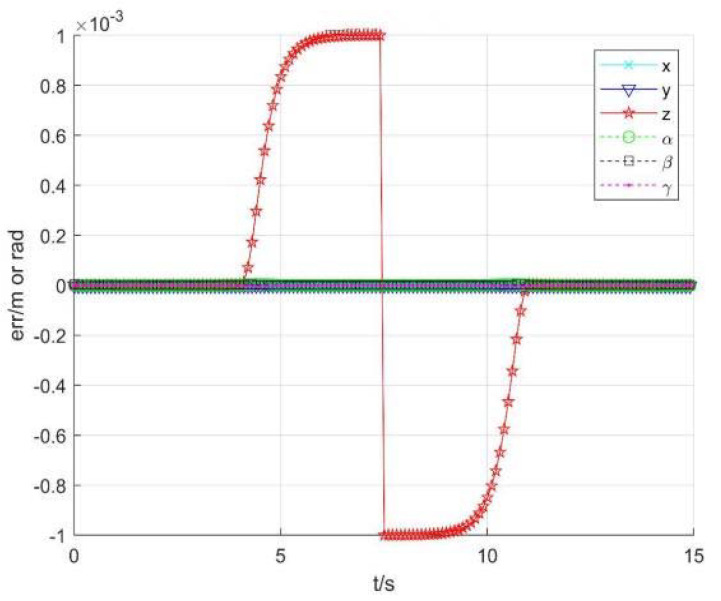
The tracking errors of position and orientation.

Figure 13 and Figure 14 show the position and speed change curve of each joint, respectively. Figure 15 shows the running speed of the robot end, Figure 16 shows each item changes of constant *b*, and Figure 17 shows the tracking errors of position and orientation.

## 5. Results Discussion

From the simulation results in Section 4.1, it is noted that both accuracies can meet the requirement of inverse kinematics. The average calculation time of using the numerical method to calculate the inverse kinematics solution is 0.0021 s, and using the analytical method is 0.0014 s. The calculation time of two methods is in the same order of magnitude. However, when the robot is in the singular configuration, the numerical method takes three times more time than the numerical method due to more iterations needed.

Figure 7, Figure 8 and Figure 9 show variation curves of joint position, velocity, and tracking error with time under no avoiding measures. It is obvious that the robot enters the singular region in about 4.65 s. At this time, the angle value of joint 4 is close to π  and meets the condition of external singularity. Due to the ill condition of the Jacobian matrix, the obtained joint angle has a sudden change, and the joint speed is particularly huge, which is impossible for the motor to realize in practice. Meanwhile, the robot tracking error end pose becomes larger after entering the singular region, since the upper motor speed cannot be tracked in practical operation.

From Figure 10, Figure 11 and Figure 12, it can be seen that with damping Jacobian matrix (21), the joint angle changes smoothly, and the whole joint speed can be below 1.5 rad/s. After entering the singular region at about 4.65 s, the pose tracking error will also increase to a certain extent. However, the avoidance method has a certain effect on limiting joint speeds, it is too sensitive to the selection of parameters. Hence, it is necessary to design and select appropriate control parameters for different task requirements.

Figure 13, Figure 14, Figure 15, Figure 16 and Figure 17 show variation curves of joint position, velocity, end position, and tracking error with singular consistency to avoid singularity. It can be seen that the robot enters the singular region at about 4.1 s. By changing constant value $b_{i}$, which makes the following det *J_i_* smaller, the desired robot end speed d*p* are forced to change (Figure 15), to limit the speed of each joint (Figure 14). Compared with the singularity avoidance methods in Section 3.1, the joint operation is more stable, and the operation speed of each joint can also transit smoothly when entering into singularity region. Moreover, it will not cause an error deviating from desired trajectory. Therefore, when the robot end velocity is constant, the damping least square avoidance method is selected, while the singular consistency avoidance method is more appropriate when the end velocity changes.

## 6. Conclusions

The emergence of co-bots is an important supplement to traditional industrial robots, especially for new potential users such as small and medium-sized enterprises and increasingly complex control tasks in 3C electronic products, which determines that co-bots should have characteristics of safe use, simple operation, and convenient deployment. Taking the JACO2 robot as a carrier, this paper provides research on singularity analysis and avoidance strategies to realize safe control and smooth operation of a co-bot in the increasingly complex working environment and man–machine intelligent interactive control. The research results are summarized as follows.

The instability and potential safety hazards of the robot in the singular region are analyzed in detail. The conditions for singularity of the robot are deduced by means of Jacobian matrix separation. Then, the singular configuration conditions are analyzed in detail by means of block analysis of robot wrist Jacobian matrix, which can be divided into three singular types: internal singularity, external singularity, and wrist singularity. For different types of singularity, three singularity avoidance schemes are realized based on robot redundancy and different control tasks. This will ensure safe and stable operation of the co-bot in the whole workspace, and it eliminates safety problems in the man–machine cooperation.

In the future work, it is necessary to study some security issues through other sensors to ensure safety from the perspective of smooth operation, for example, replanning tasks to avoid collision, and adjusting the robot running state according to the working state of the operator. In addition, compliance control can also be studied to improve the robot adaptability with complex industrial tasks.

## Figures and Tables

**Table 1:** D-H parameters of the JACO2 robot.

i	$\alpha_{i-1}$ (rad)	$a_{i-1}$ (m)	$d_{i}$ (m)	$\theta_{i}$ **(rad)**
1	π/2	0	−0.2755	$\theta_{1}$
2	π/2	0	0	$\theta_{2}$
3	π/2	0	−0.41	$\theta_{3}$
4	π/2	0	−0.0098	$\theta_{4}$
5	π/2	0	−0.3111	$\theta_{5}$
6	π/2	0	0	$\theta_{6}$
7	π	0	−0.2638	$\theta_{7}$

**Table 2:** The condition in which the determinant of the Jw11 cofactor of each order is 0.

Number	Condition	*M*_1_	*M*_2_	*M*_3_	*M*_4_
1	$s_{4}=0$	**√**	**√**	**√**	**√**
2	$d_{3}s_{2}-d_{5}s_{2}c_{4}+c_{2}d_{5}c_{3}s_{4}+c_{2}d_{4}s_{3}=0$	**√**	**√**		
3	$s_{2}=0$	**√**		**√**	
4	$c_{3}d_{4}-d_{5}s_{3}s_{4}=0$	**√**		**√**	
5	$d_{4}s_{3}+d_{5}s_{3}s_{4}=0$	**√**			**√**

**Table 3:** The position singularity condition.

Number	Condition Combination in Table 2 (Serial Number)	Simplified Conditions
1	1	$s_{4}=0$
2	2 + 3 + 5	$s_{2}=0$ $,\;d_{4}s_{3}+d_{5}s_{3}s_{4}=0$
3	2 + 4 + 5	Not established (4 and 5 cannot be met at the same time).

**Table 4:** The robot attitude singularity condition.

Number	Attitude Singularity Satisfying Condition	Note
1	$s_{6}=0\;\;\;\;\;s_{2}=0$	
2	$s_{6}=0$ $\;s_{4}=0$	The position singularity is also satisfied.
3	$s_{6}=0$ $d_{4}s_{5}+d_{3}s_{4}c_{5}=0$	

## Data Availability

Not applicable.
